# (Morpholin-4-yl)[2-(morpholin-4-yl)-3,5-dinitro­phen­yl]methanone

**DOI:** 10.1107/S1600536812000426

**Published:** 2012-01-14

**Authors:** Chao Gao, Ying Xiong, Yong Xia, Luo-Ting Yu

**Affiliations:** a State Key Laboratory of Biotherapy and Cancer Center, West China Hospital, West China Medical School, Sichuan University, Chengdu 610041, People’s Republic of China

## Abstract

In the title compound, C_15_H_18_N_4_O_7_, the morpholine rings adopt chair conformations. The benzene ring forms dihedral angles of 55.94 (7) and 63.19 (7)° with the planes through the C atoms of the two morpholine rings.

## Related literature

For the biological activity of benzamide derivatives, see: Christophe *et al.* (2009 ▶).
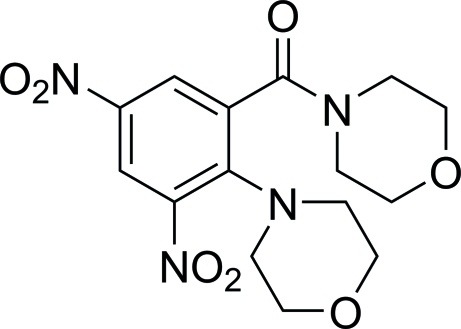


## Experimental

### 

#### Crystal data

C_15_H_18_N_4_O_7_
*M* *_r_* = 366.33
Monoclinic, 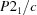

*a* = 10.2640 (4) Å
*b* = 21.5488 (7) Å
*c* = 8.0061 (3) Å
β = 108.587 (4)°
*V* = 1678.40 (10) Å^3^
*Z* = 4
Mo *K*α radiation
μ = 0.12 mm^−1^
*T* = 293 K
0.38 × 0.35 × 0.35 mm

#### Data collection

Oxford Diffraction Xcalibur Eos diffractometer
Absorption correction: multi-scan (*CrysAlis PRO*; Oxford Diffraction, 2006 ▶) *T*_min_ = 0.994, *T*_max_ = 1.000
7068 measured reflections
3422 independent reflections
2440 reflections with *I* > 2σ(*I*)
*R*_int_ = 0.017

#### Refinement

*R*[*F* ^2^ > 2σ(*F* ^2^)] = 0.044
*wR*(*F* ^2^) = 0.110
*S* = 1.03
3422 reflections
235 parameters
H-atom parameters constrained
Δρ_max_ = 0.16 e Å^−3^
Δρ_min_ = −0.20 e Å^−3^

### 

Data collection: *CrysAlis PRO* (Oxford Diffraction, 2006 ▶); cell refinement: *CrysAlis PRO*; data reduction: *CrysAlis PRO*; program(s) used to solve structure: *SHELXS97* (Sheldrick, 2008 ▶); program(s) used to refine structure: *SHELXL97* (Sheldrick, 2008 ▶); molecular graphics: *OLEX2* (Dolomanov *et al.*, 2009 ▶); software used to prepare material for publication: *OLEX2*.

## Supplementary Material

Crystal structure: contains datablock(s) I, global. DOI: 10.1107/S1600536812000426/nc2263sup1.cif

Structure factors: contains datablock(s) I. DOI: 10.1107/S1600536812000426/nc2263Isup2.hkl

Supplementary material file. DOI: 10.1107/S1600536812000426/nc2263Isup3.cml

Additional supplementary materials:  crystallographic information; 3D view; checkCIF report

## Figures and Tables

**Fig. 1.**
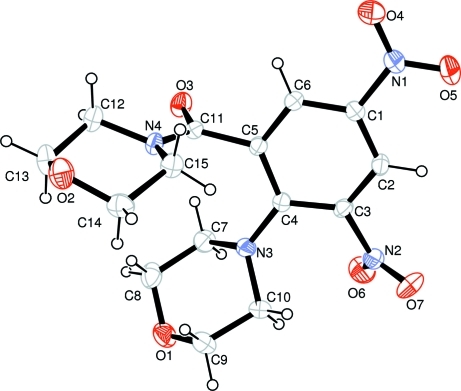
The molecular structure of the title compound with labeling and displacement ellipsoids drawn at the 30% probability level.

## supplementary crystallographic information

### Comment

Benzamide derivatives are of great importance owing to their antibacterial properties (Christophe *et al.*, 2009). The title compound is one of the key intermediates in our synthetic investigations of antibacterial drugs. Therefore, its crystal structure was determined.

In the crystal structure of the title compound, C_15_H_18_N_4_O_7_, the morpholine rings adopt a chair conformation. The benzene ring forms dihedral angles of 55.94 (7)° and 63.19 (7)° with the two morpholine rings.

### Experimental

3.42 g (12.9 mmol) of 2-chloro-3,5-dinitrobenzoyl chloride was added to a solution of 2.36 g (27.1 mmol) morpholine in 20 ml of dichloromethane and 1.82 g (17.9 mmol) triethylamine. The mixture was stirred for 2 h at room temperature extracted with water and dichloromethane and the organic solvent was evaporated and the title compound was recrystallized from ethanol (yield 4.25 g, 90%). Crystals suitable for X-ray analysis were obtained by slow evaporation of the solvent from a solution of the title compound in acetonitrile.

### Refinement

All H atoms were positioned geometrically (C—H = 0.93–0.97 Å) and refined using a riding model, with *U*_iso_(H) = 1.2*U*_eq_(C).

### Crystal data

**Table:**

C_15_H_18_N_4_O_7_	*F*(000) = 768
*M**_r_* = 366.33	*D*_x_ = 1.450 Mg m^−^^3^
Monoclinic, *P*2_1_/*c*	Mo *K*α radiation, λ = 0.7107 Å
*a* = 10.2640 (4) Å	Cell parameters from 2613 reflections
*b* = 21.5488 (7) Å	θ = 3.0–29.0°
*c* = 8.0061 (3) Å	µ = 0.12 mm^−^^1^
β = 108.587 (4)°	*T* = 293 K
*V* = 1678.40 (10) Å^3^	Block, yellow
*Z* = 4	0.38 × 0.35 × 0.35 mm

### Data collection

**Table:**

Oxford Diffraction Xcalibur Eos diffractometer	3422 independent reflections
Radiation source: Enhance (Mo) X-ray Source	2440 reflections with *I* > 2σ(*I*)
graphite	*R*_int_ = 0.017
Detector resolution: 16.0874 pixels mm^-1^	θ_max_ = 26.4°, θ_min_ = 3.0°
ω scans	*h* = −12→7
Absorption correction: multi-scan (CrysAlis PRO; Oxford Diffraction, 2006)	*k* = −26→18
*T*_min_ = 0.994, *T*_max_ = 1.000	*l* = −9→10
7068 measured reflections	

### Refinement

**Table:**

Refinement on *F*^2^	Primary atom site location: structure-invariant direct methods
Least-squares matrix: full	Secondary atom site location: difference Fourier map
*R*[*F*^2^ > 2σ(*F*^2^)] = 0.044	Hydrogen site location: inferred from neighbouring sites
*wR*(*F*^2^) = 0.110	H-atom parameters constrained
*S* = 1.03	*w* = 1/[σ^2^(*F*_o_^2^) + (0.0426*P*)^2^ + 0.3019*P*] where *P* = (*F*_o_^2^ + 2*F*_c_^2^)/3
3422 reflections	(Δ/σ)_max_ < 0.001
235 parameters	Δρ_max_ = 0.16 e Å^−^^3^
0 restraints	Δρ_min_ = −0.20 e Å^−^^3^

### Special details

**Table:**

Experimental. CrysAlisPro (Oxford Diffraction, 2006). Agilent Technologies, Version 1.171.35.19 (release 27-10-2011 CrysAlis171 .NET) (compiled Oct 27 2011,15:02:11) Empirical absorption correction using spherical harmonics, implemented in SCALE3 ABSPACK scaling algorithm.
Geometry. All e.s.d.'s (except the e.s.d. in the dihedral angle between two l.s. planes) are estimated using the full covariance matrix. The cell e.s.d.'s are taken into account individually in the estimation of e.s.d.'s in distances, angles and torsion angles; correlations between e.s.d.'s in cell parameters are only used when they are defined by crystal symmetry. An approximate (isotropic) treatment of cell e.s.d.'s is used for estimating e.s.d.'s involving l.s. planes.
Refinement. Refinement of *F*^2^ against ALL reflections. The weighted *R*-factor *wR* and goodness of fit *S* are based on *F*^2^, conventional *R*-factors *R* are based on *F*, with *F* set to zero for negative *F*^2^. The threshold expression of *F*^2^ > σ(*F*^2^) is used only for calculating *R*-factors(gt) *etc*. and is not relevant to the choice of reflections for refinement. *R*-factors based on *F*^2^ are statistically about twice as large as those based on *F*, and *R*- factors based on ALL data will be even larger.

### Fractional atomic coordinates and isotropic or equivalent isotropic displacement parameters (Å^2^)

**Table:**

	*x*	*y*	*z*	*U*_iso_*/*U*_eq_	
O1	0.38171 (14)	0.03743 (7)	0.58276 (17)	0.0608 (4)	
O2	0.58939 (14)	0.11924 (7)	0.02632 (18)	0.0588 (4)	
O3	0.27069 (12)	0.25026 (6)	0.21720 (17)	0.0468 (3)	
O4	−0.13992 (17)	0.22884 (9)	−0.4060 (2)	0.0884 (6)	
O5	−0.30029 (15)	0.16911 (8)	−0.38749 (19)	0.0723 (5)	
O6	−0.11367 (15)	0.07133 (8)	0.3247 (2)	0.0697 (5)	
O7	−0.17948 (18)	0.01161 (7)	0.0962 (2)	0.0825 (5)	
N1	−0.18359 (16)	0.18932 (8)	−0.3309 (2)	0.0500 (4)	
N2	−0.12006 (16)	0.05713 (8)	0.1748 (2)	0.0510 (4)	
N3	0.17106 (14)	0.09335 (6)	0.30645 (17)	0.0375 (4)	
N4	0.36454 (13)	0.17369 (6)	0.09991 (17)	0.0342 (3)	
C1	−0.09372 (17)	0.16569 (8)	−0.1628 (2)	0.0361 (4)	
C2	−0.14151 (17)	0.12014 (8)	−0.0794 (2)	0.0373 (4)	
H2	−0.2291	0.1036	−0.1289	0.045*	
C3	−0.05571 (17)	0.09943 (8)	0.0810 (2)	0.0342 (4)	
C4	0.07972 (16)	0.12074 (7)	0.1600 (2)	0.0307 (4)	
C5	0.12146 (16)	0.16958 (7)	0.0703 (2)	0.0306 (4)	
C6	0.03563 (17)	0.19131 (8)	−0.0891 (2)	0.0357 (4)	
H6	0.0648	0.2232	−0.1471	0.043*	
C7	0.25205 (19)	0.12947 (9)	0.4592 (2)	0.0464 (5)	
H7A	0.2059	0.1302	0.5476	0.056*	
H7B	0.2613	0.1719	0.4238	0.056*	
C8	0.3922 (2)	0.10043 (11)	0.5348 (3)	0.0563 (6)	
H8A	0.4402	0.1024	0.4485	0.068*	
H8B	0.4451	0.1236	0.6379	0.068*	
C9	0.3045 (2)	0.00286 (10)	0.4328 (3)	0.0570 (5)	
H9A	0.2990	−0.0400	0.4668	0.068*	
H9B	0.3515	0.0037	0.3452	0.068*	
C10	0.16127 (19)	0.02827 (8)	0.3529 (3)	0.0460 (5)	
H10A	0.1125	0.0047	0.2485	0.055*	
H10B	0.1110	0.0249	0.4366	0.055*	
C11	0.25990 (16)	0.20097 (8)	0.1370 (2)	0.0317 (4)	
C12	0.49371 (17)	0.20777 (9)	0.1316 (3)	0.0456 (5)	
H12A	0.5083	0.2348	0.2329	0.055*	
H12B	0.4886	0.2334	0.0301	0.055*	
C13	0.61214 (19)	0.16320 (11)	0.1647 (3)	0.0594 (6)	
H13A	0.6957	0.1862	0.1754	0.071*	
H13B	0.6246	0.1416	0.2749	0.071*	
C14	0.4726 (2)	0.08302 (9)	0.0182 (3)	0.0493 (5)	
H14A	0.4871	0.0625	0.1305	0.059*	
H14B	0.4603	0.0513	−0.0714	0.059*	
C15	0.34554 (18)	0.12219 (8)	−0.0241 (2)	0.0419 (4)	
H15A	0.3240	0.1383	−0.1429	0.050*	
H15B	0.2688	0.0968	−0.0189	0.050*	

### Atomic displacement parameters (Å^2^)

**Table:**

	*U*^11^	*U*^22^	*U*^33^	*U*^12^	*U*^13^	*U*^23^
O1	0.0474 (8)	0.0784 (11)	0.0501 (8)	0.0055 (7)	0.0066 (7)	0.0204 (8)
O2	0.0462 (8)	0.0699 (10)	0.0680 (9)	0.0094 (7)	0.0290 (7)	−0.0044 (8)
O3	0.0433 (7)	0.0360 (7)	0.0618 (8)	−0.0055 (6)	0.0177 (6)	−0.0154 (6)
O4	0.0654 (11)	0.1098 (14)	0.0752 (11)	−0.0167 (10)	0.0017 (9)	0.0537 (11)
O5	0.0423 (8)	0.0936 (12)	0.0631 (9)	−0.0114 (8)	−0.0086 (7)	0.0163 (8)
O6	0.0582 (10)	0.0991 (13)	0.0616 (10)	0.0069 (8)	0.0327 (8)	0.0293 (9)
O7	0.0735 (11)	0.0540 (10)	0.1152 (14)	−0.0298 (9)	0.0236 (10)	0.0129 (10)
N1	0.0393 (9)	0.0576 (11)	0.0462 (9)	−0.0007 (8)	0.0038 (7)	0.0108 (8)
N2	0.0357 (9)	0.0519 (11)	0.0666 (11)	0.0001 (8)	0.0183 (8)	0.0201 (9)
N3	0.0382 (8)	0.0352 (8)	0.0352 (7)	−0.0014 (6)	0.0062 (6)	0.0034 (6)
N4	0.0290 (7)	0.0343 (8)	0.0393 (8)	−0.0048 (6)	0.0111 (6)	−0.0061 (6)
C1	0.0323 (9)	0.0381 (10)	0.0361 (9)	0.0012 (7)	0.0084 (7)	0.0040 (8)
C2	0.0268 (8)	0.0387 (10)	0.0446 (10)	−0.0034 (7)	0.0090 (7)	−0.0009 (8)
C3	0.0336 (9)	0.0305 (9)	0.0423 (9)	−0.0025 (7)	0.0176 (8)	0.0024 (8)
C4	0.0300 (8)	0.0306 (9)	0.0325 (8)	0.0006 (7)	0.0114 (7)	−0.0032 (7)
C5	0.0295 (8)	0.0279 (8)	0.0356 (8)	−0.0003 (7)	0.0122 (7)	−0.0014 (7)
C6	0.0332 (9)	0.0343 (9)	0.0406 (9)	−0.0020 (7)	0.0131 (7)	0.0048 (8)
C7	0.0502 (11)	0.0525 (12)	0.0341 (9)	−0.0053 (9)	0.0100 (8)	−0.0001 (8)
C8	0.0444 (11)	0.0781 (16)	0.0420 (11)	−0.0116 (11)	0.0076 (9)	0.0061 (11)
C9	0.0494 (12)	0.0536 (13)	0.0646 (13)	0.0120 (10)	0.0134 (10)	0.0161 (11)
C10	0.0458 (11)	0.0374 (10)	0.0510 (11)	0.0012 (8)	0.0102 (9)	0.0137 (9)
C11	0.0320 (9)	0.0291 (9)	0.0334 (8)	−0.0023 (7)	0.0097 (7)	0.0018 (7)
C12	0.0339 (10)	0.0499 (12)	0.0546 (11)	−0.0099 (8)	0.0163 (8)	−0.0076 (9)
C13	0.0320 (10)	0.0768 (16)	0.0670 (13)	0.0005 (10)	0.0125 (10)	−0.0057 (12)
C14	0.0572 (13)	0.0440 (11)	0.0521 (11)	0.0086 (10)	0.0252 (10)	0.0016 (9)
C15	0.0421 (10)	0.0372 (10)	0.0477 (10)	−0.0012 (8)	0.0164 (8)	−0.0102 (8)

### Geometric parameters (Å, °)

**Table:**

O1—C8	1.424 (2)	C5—C6	1.381 (2)
O1—C9	1.420 (2)	C5—C11	1.509 (2)
O2—C13	1.419 (2)	C6—H6	0.9300
O2—C14	1.414 (2)	C7—H7A	0.9700
O3—C11	1.2275 (19)	C7—H7B	0.9700
O4—N1	1.208 (2)	C7—C8	1.507 (3)
O5—N1	1.218 (2)	C8—H8A	0.9700
O6—N2	1.220 (2)	C8—H8B	0.9700
O7—N2	1.219 (2)	C9—H9A	0.9700
N1—C1	1.460 (2)	C9—H9B	0.9700
N2—C3	1.465 (2)	C9—C10	1.507 (2)
N3—C4	1.379 (2)	C10—H10A	0.9700
N3—C7	1.464 (2)	C10—H10B	0.9700
N3—C10	1.463 (2)	C12—H12A	0.9700
N4—C11	1.338 (2)	C12—H12B	0.9700
N4—C12	1.465 (2)	C12—C13	1.505 (3)
N4—C15	1.460 (2)	C13—H13A	0.9700
C1—C2	1.363 (2)	C13—H13B	0.9700
C1—C6	1.384 (2)	C14—H14A	0.9700
C2—H2	0.9300	C14—H14B	0.9700
C2—C3	1.379 (2)	C14—C15	1.499 (2)
C3—C4	1.408 (2)	C15—H15A	0.9700
C4—C5	1.415 (2)	C15—H15B	0.9700
			
C9—O1—C8	109.99 (14)	C7—C8—H8B	109.4
C14—O2—C13	109.29 (14)	H8A—C8—H8B	108.0
O4—N1—O5	122.95 (17)	O1—C9—H9A	109.2
O4—N1—C1	118.63 (16)	O1—C9—H9B	109.2
O5—N1—C1	118.41 (16)	O1—C9—C10	111.99 (17)
O6—N2—C3	117.18 (17)	H9A—C9—H9B	107.9
O7—N2—O6	124.81 (18)	C10—C9—H9A	109.2
O7—N2—C3	117.97 (18)	C10—C9—H9B	109.2
C4—N3—C7	122.26 (14)	N3—C10—C9	108.64 (15)
C4—N3—C10	122.91 (14)	N3—C10—H10A	110.0
C10—N3—C7	111.36 (14)	N3—C10—H10B	110.0
C11—N4—C12	119.16 (14)	C9—C10—H10A	110.0
C11—N4—C15	122.84 (13)	C9—C10—H10B	110.0
C15—N4—C12	114.60 (13)	H10A—C10—H10B	108.3
C2—C1—N1	118.85 (15)	O3—C11—N4	123.45 (15)
C2—C1—C6	121.64 (15)	O3—C11—C5	119.39 (15)
C6—C1—N1	119.47 (15)	N4—C11—C5	117.13 (14)
C1—C2—H2	121.1	N4—C12—H12A	109.6
C1—C2—C3	117.78 (15)	N4—C12—H12B	109.6
C3—C2—H2	121.1	N4—C12—C13	110.25 (16)
C2—C3—N2	114.83 (15)	H12A—C12—H12B	108.1
C2—C3—C4	123.97 (15)	C13—C12—H12A	109.6
C4—C3—N2	120.94 (15)	C13—C12—H12B	109.6
N3—C4—C3	123.28 (15)	O2—C13—C12	111.48 (16)
N3—C4—C5	121.07 (14)	O2—C13—H13A	109.3
C3—C4—C5	115.44 (14)	O2—C13—H13B	109.3
C4—C5—C11	123.82 (14)	C12—C13—H13A	109.3
C6—C5—C4	120.93 (14)	C12—C13—H13B	109.3
C6—C5—C11	115.25 (14)	H13A—C13—H13B	108.0
C1—C6—H6	120.0	O2—C14—H14A	109.4
C5—C6—C1	120.07 (15)	O2—C14—H14B	109.4
C5—C6—H6	120.0	O2—C14—C15	111.31 (16)
N3—C7—H7A	109.8	H14A—C14—H14B	108.0
N3—C7—H7B	109.8	C15—C14—H14A	109.4
N3—C7—C8	109.28 (16)	C15—C14—H14B	109.4
H7A—C7—H7B	108.3	N4—C15—C14	110.89 (15)
C8—C7—H7A	109.8	N4—C15—H15A	109.5
C8—C7—H7B	109.8	N4—C15—H15B	109.5
O1—C8—C7	111.10 (16)	C14—C15—H15A	109.5
O1—C8—H8A	109.4	C14—C15—H15B	109.5
O1—C8—H8B	109.4	H15A—C15—H15B	108.0
C7—C8—H8A	109.4		
			
O1—C9—C10—N3	57.5 (2)	C4—N3—C10—C9	144.78 (16)
O2—C14—C15—N4	−54.3 (2)	C4—C5—C6—C1	0.6 (2)
O4—N1—C1—C2	−178.60 (18)	C4—C5—C11—O3	−97.7 (2)
O4—N1—C1—C6	3.5 (3)	C4—C5—C11—N4	84.29 (19)
O5—N1—C1—C2	2.8 (3)	C6—C1—C2—C3	−0.9 (3)
O5—N1—C1—C6	−175.07 (18)	C6—C5—C11—O3	83.08 (19)
O6—N2—C3—C2	−124.82 (18)	C6—C5—C11—N4	−94.98 (18)
O6—N2—C3—C4	49.6 (2)	C7—N3—C4—C3	−131.89 (17)
O7—N2—C3—C2	52.9 (2)	C7—N3—C4—C5	53.6 (2)
O7—N2—C3—C4	−132.64 (19)	C7—N3—C10—C9	−55.9 (2)
N1—C1—C2—C3	−178.74 (15)	C8—O1—C9—C10	−59.5 (2)
N1—C1—C6—C5	179.70 (15)	C9—O1—C8—C7	59.2 (2)
N2—C3—C4—N3	16.1 (2)	C10—N3—C4—C3	25.3 (2)
N2—C3—C4—C5	−169.09 (15)	C10—N3—C4—C5	−149.27 (16)
N3—C4—C5—C6	171.26 (15)	C10—N3—C7—C8	56.40 (19)
N3—C4—C5—C11	−8.0 (2)	C11—N4—C12—C13	153.48 (16)
N3—C7—C8—O1	−57.6 (2)	C11—N4—C15—C14	−154.09 (16)
N4—C12—C13—O2	54.2 (2)	C11—C5—C6—C1	179.86 (15)
C1—C2—C3—N2	171.64 (16)	C12—N4—C11—O3	−9.5 (2)
C1—C2—C3—C4	−2.6 (3)	C12—N4—C11—C5	168.46 (14)
C2—C1—C6—C5	1.9 (3)	C12—N4—C15—C14	47.0 (2)
C2—C3—C4—N3	−170.01 (15)	C13—O2—C14—C15	62.6 (2)
C2—C3—C4—C5	4.8 (2)	C14—O2—C13—C12	−62.8 (2)
C3—C4—C5—C6	−3.7 (2)	C15—N4—C11—O3	−167.52 (16)
C3—C4—C5—C11	177.09 (14)	C15—N4—C11—C5	10.4 (2)
C4—N3—C7—C8	−144.08 (16)	C15—N4—C12—C13	−46.8 (2)

## References

[bb1] Christophe, T., Jackson, M., Jeon, H. K., Fenistein, D., Contreras-Dominguez, M., Kim, J., Genovesio, A., Carralot, J. P., Ewann, F., Kim, E. H., Lee, S. Y., Kang, S., Seo, M. J., Park, E. J., Skovierova, H., Pham, H., Riccardi, G., Nam, J. Y., Marsollier, L., Kempf, M., Joly-Guillou, M. L., Oh, T., Shin, W. K., No, Z., Nehrbass, U., Brosch, R., Cole, S. T. & Brodin, P. (2009). *PLoS Pathog.* **5**, 1–10. 10.1371/journal.ppat.1000645 PMC2763345 19876393

[bb2] Dolomanov, O. V., Bourhis, L. J., Gildea, R. J., Howard, J. A. K. & Puschmann, H. (2009). *J. Appl. Cryst.* **42**, 339–341.

[bb3] Oxford Diffraction (2006). *CrysAlis PRO* Oxford Diffraction Ltd, Abingdon, England.

[bb4] Sheldrick, G. M. (2008). *Acta Cryst.* A**64**, 112–122. 10.1107/S0108767307043930 18156677

